# Association between C282Y and H63D mutations of the HFE gene with hepatocellular carcinoma in European populations: a meta-analysis

**DOI:** 10.1186/1756-9966-29-18

**Published:** 2010-03-02

**Authors:** Fei Jin, Li-Shuai Qu, Xi-Zhong Shen

**Affiliations:** 1 Department of Gastroenterology, Zhongshan Hospital, Shanghai Medical College, Fudan University, Shanghai 200032, China

## Abstract

**Background:**

Hereditary hemochromatosis (HH) is an autosomal recessive disorder mainly associated with homozygosity for the C282Y and H63D mutations in the hemochromatosis (HFE) gene. The reports about the C282Y and H63D mutations and hepatocellular carninoma (HCC) were controversial. To clarify the relationship between C282Y and H63D mutations and HCC, a meta-analysis including nine studies (1102 HCC cases and 3766 controls, mainly came from European populations) was performed.

**Methods:**

The association was measured using random-effect (RE) or fixed-effect (FE) odds ratios (ORs) combined with 95% confidence intervals (CIs) according to the studies' heterogeneity.

**Results:**

Meta-analysis of nine studies showed that Y allele of C282Y was associated with HCC risk: RE OR reached 1.50 (95%CI: 1.05-2.14, p for heterogeneity = 0.02, I^2 ^= 0.57). Subgroup analysis of seven studies also showed Y allele was associated with HCC risk in healthy populations: RE OR reached 1.61 (95%CI: 1.08-2.39, p for heterogeneity = 0.04, I^2 ^= 0.55). We further did subgroup analysis in alcoholic liver cirrhosis (LC) patients of four studies (224 cases and 380 controls) and found that both the dominant model and Y allele of C282Y were associated with HCC risk (FE OR reached 4.06, 95%CI: 2.08-7.92 and 3.41, 95%CI: 1.81-6.41, respectively). There was no distinct heterogeneity among the studies (I^2 ^= 0). Sensitivity analyses showed the results were robust in the subgroup analysis of alcoholic LC patients.

**Conclusions:**

C282Y mutation was associated with HCC in European alcoholic LC patients.

## Background

Hepatocellular carninoma (HCC) is the fifth most common cancer in the world and the third most common cause of cancer mortality [1]. Hereditary hemochromatosis (HH) is an autosomal recessive genetic condition in which excess iron is absorbed by the intestine and deposited throughout the body [2]. If untreated, affected individuals may accumulate excess iron over the many years of their adult life, and this causes progressive tissue damage [3]. It has been reported that HH may result in many diseases, including liver disease (fibrosis, cirrhosis, and hepatocellular carcinoma). Some studies reported that liver disease was the most common cause of death of patients with HH [4,5].

In 1996, Feder and colleagues [6] showed that homozygosity for mutation (C282Y, G>A, rs1800562) in the HFE gene was responsible for the majority of cases of typical phenotypic HH. The frequency of the second variant (H63D, C>G, rs1799945) is also increased in HH patients, but its penetrance is low. From then on, HFE gene has been postulated as a candidate gene of HCC. Some studies [7-16] demonstrated that C282Y or H63D increased the risk of HCC, while some [17-19] gave negative results. Some large scale cohort studies [20,21] also showed that HFE gene mutation penetrance was low and did not increase the likelihood of death from any cause among the C282Y homozygotes compared with subjects who had no C282Y mutation.

However, the estimates in these cohort studies were conservative in the sense that in the cohort study period, a proportion of HH patients had received phlebotomy treatment. As a result, the role of C282Y and H63D mutations in HCC occurrence still merits study. To clarify the relationship between HFE C282Y and H63D mutations and HCC, a meta-analysis was performed.

## Methods

### Study identification and selection

Eligible studies were identified by searching the databases of PubMed and ISI Web of Knowledge for relevant reports published before May 2009. The search criteria "c282y OR h63d" and "liver cancer OR hepatocellular carcinoma" were used. We also searched reports and dissection databases published in the Chinese Biomedical database (CBM), China National Knowledge Infrastructure (CNKI), and Wan Fang (Chinese) database to collect articles of case-control studies or cohort studies on associations between HFE mutations and susceptibility to HCC before May 2009. The reference lists of the retrieved articles were also reviewed to identify additional articles missed by the above search.

Studies were selected if (1) there were available data for at least one of the C282Y and H63D two mutations with risk of HCC using a case-control or cohort design; (2) HCC cases were diagnosed by histopathological biopsy or by elevated AFP and distinct iconography changes (CT, MRI, and B ultrasonography); (3) control subjects were free of cancer. Controls could be composed of healthy subjects, chronic liver disease (CLD), including chronic hepatitis (CH) and LC. CLD was either histologically proven or diagnosed based on concordant clinical, biological, and morphological criteria. Review articles and articles that did not provide genotype data were excluded.

### Data extraction and synthesis

The following information was extracted from each study: first author's surname, year of publication, ethnicity of study population, country where study was conducted, and the number of cases and controls for each C282Y and H63D genotype. When specific results were not reported, we used available tabular data to calculate them.

### Statistical methods

To compare the odds ratio (OR) on the same baseline, we used crude OR to conduct the meta-analysis. The effect of association was indicated as crude OR with the corresponding 95% confidence intervals (CIs). Because of relatively small sample sizes of individual studies and low frequency of variant alleles and the practical clinical value, we performed meta-analysis only in two models: dominant model (YY+CY vs. CC or DD+HD vs. HH) and allele contrast (Y vs. C or D vs. H). The pooled OR was estimated using the FE model (DerSimonian & Laird) [22]. The heterogeneity between studies was tested using the Q statistic [23]. If P < 0.10, the heterogeneity was considered statistically significant, and the RE model was then used. Heterogeneity was also quantified using the I^2 ^metric, which is independent of the number of studies in the meta-analysis (I^2 ^< 25% = no heterogeneity; I^2 ^= 25-50% = moderate heterogeneity; I^2 ^> 50% = large or extreme heterogeneity) [24]. The potential small-study bias was tested using the Egger regression test asymmetry [25] and the Begg's test for funnel plot, which is based on Kendall's tau [26]. Sensitivity analysis was performed by omitting one study at a time to assess the influence of individual studies on meta-analysis. The distribution of the genotypes in the control group was tested for Hardy-Weinberg equilibrium using a goodness-of-fit Chi-square test.

All analyses above were conducted using the STATA version 10.0 software (Stata Corp, College Station, Texas). All P-values were two-sided. A p value less than 0.05 was considered statistically significant.

The statistical power was calculated using the PS software [27]. In order to assess the reliability of the positive association, we calculated false positive report probability (FPRP) [28]. An Excel spreadsheet to calculate FPRP is included with the online material http://jncicancerspectrum.oupjournals.org/jnci/content/vol96/issue6. If FPRP < 0.20, we think the association is reliable. Given that the gene mutation was regarded as causal, we used population-attributable risk (PAR) to refer to the proportion of disease risk in a population that can be attributed to the causal effects of the risk allele. PAR can be assessed by using the formula [29].

## Results

### Eligible studies

By searching data, we found that 15 articles [7-19,30,31] used case-control or cohort design to explore the relationship between HFE mutation and HCC. Six studies [7,9,13,18,19,30] were excluded either because of insufficient numbers of samples or because they did not provide concrete genotype data. Altogether, nine studies [8,10-12,14-17,31] which contained 1102 cases and 3766 controls met the inclusion criteria and were included in the final analysis. Eight studies were published in English and one study was published in Spanish[16]. Five studies [8,12,14,16,17] used peripheral blood leucocytes, two studies used liver tissue [10,31]and two studies used both blood and liver tissue [11,15] to extract genome DNA. All studies used validated methods to genotype the C282Y and or H63D mutation. Seven studies [7-9,11,12,14,16,17,31] used PCR-RFLP, one study [10] used the Taqman method, and one study [15] used PCR combined with 3'minor groove binding group (MGB) probe fluorescent hybridization. Of the nine studies, eight studies (including 958 cases and 2258 controls) also explored the relationship between H63D and HCC (Table 1).

**Table 1:** Main characteristics of all studies included in the meta-analysis

					C282Y						H63D					
Author	Year	Country	Study design	Cases/Controls	cases			controls			cases			controls		
					CC	CY	YY	CC	CY	YY	HH	HD	DD	HH	HD	DD
Ezzkiouri	2008	Maroc	Case-control	96/222	95	1	0	219	3	0	59	34	3	160	60	2
Nahon	2008	France	Cohort	103/198	91	12	0	180	18	0	75	28	0	149	49	0
Repero	2007	Spain	Case-control	196/181	183	12	1	158	23	0	102	85	9	124	52	5
Willis	2005	England	Case-control	144/1508	119	17	8	1331	168	9						
Hellerbrand	2003	Germany	Case-control	137/233	120	17	0	223	10	0	108	27	2	177	52	4
Cauza	2003	Austria	Case-control	162/671	139	18	5	603	63	5	128	31	3	529	133	9
Boige	2003	France	Case-control	133/100	126	7	0	93	6	1	92	41	0	59	40	1
Lauret	2002	Spain	Case-control	77/359	65	12	0	337	22	0	52	25	0	234	92	33
Beckman	2000	Sweden	Case-control	54/294	43	10	1	255	38	1	37	17	0	229	59	6

All studies were published between 2000 and 2008. In all studies, the cases were histologically confirmed or diagnosed by elevated AFP and distinct iconography changes (CT, MRI, and B ultrasonography). All the controls were free of cancer. The characteristics of the controls varied across studies: five studies [8,11,12,15,17] used CLD patients (four studies used LC patients as controls and one study used HCV CH as controls) and seven studies [8,10-12,14,16,31] included healthy population as controls. LC was diagnosed according to clinical and iconography changes. LC was classified as alcoholic LC and viral LC according to clinical and virology data. HCV was the main etiology of CLD in five studies and only a small proportion of CLD was caused by HBV. Studies were conducted in different ethnicities, mainly in European populations; eight studies [8,10-12,15-17,31] were conducted in populations of European ethnicity, and one study [14] was conducted in Marco Africans. The Hardy-Weinberg equilibrium (HWE) p values of C282Y or H63D genotypes were below 0.05 in the controls of three studies [8,12,17]. The disequilibrium might be caused by population stratification or by genotyping errors. The meta-analysis results were then assessed by excluding these studies.

### Meta-analysis results

#### C282Y

The frequency of the C282Y Y allele was 6.17% (136/2204) and 5.08% (383/7352) in cases and controls (p = 0.046), respectively, indicating that the variant allele was more frequent in cases.

**Figure 1:**
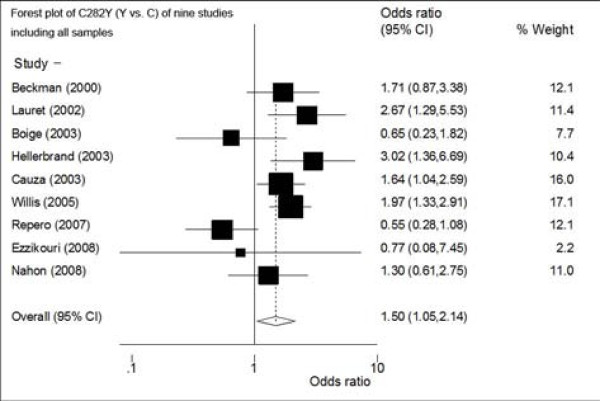
**Forest plot of the RE ORs and 95% CIs of the association between HCC and the C282Y mutation (Y vs. C) of nine studies**. The combined estimate is indicated by the diamond. The solid vertical line represents the null result.

At first, we performed the meta-analysis of nine studies including all controls to explore the association of C282Y polymorphism and HCC. Meta-analysis showed that C282Y polymorphism was associated with HCC in allele contrast model (Y vs. C): FE OR reached 1.50 (95%CI: 1.05-2.14) (Figure 1) (Table 2). There was distinct heterogeneity among studies (p for heterogeneity = 0.02, I^2 ^= 0.57). Sensitivity analysis showed that the result was not robust. There was no distinct small-study bias among the studies (Egger's p = 0.39). The meta-analysis of dominant model showed a non-significant increased risk to HCC: RE OR was 1.43 (95%CI: 0.98-2.07, p for heterogeneity = 0.02, I^2 ^= 0.55). There was no distinct small-study bias among the studies (Egger's p = 0.68).

**Table 2:** Meta-analysis results of C282Y polymorphism and HCC

	Nine studies of all samples			Seven studies of healthy controls		Four studies of alcoholic LC		Four studies of viral LC	
Genetic model	Dominant	Allele contrast	CY vs. CC	Dominant	Allele contrast	Dominant	Allele contrast	Dominant	Allele contrast
OR	1.43	**1.50**	1.31	1.46	**1.61**	**4.06**	**3.41**	0.70	0.71
95%CI	0.98-2.07	**1.05-2.14**	0.89-1.95	0.96-2.22	**1.08-2.39**	**2.08-7.92**	**1.81-6.41**	0.32-1.50	0.34-1.50
p for hetero	0.02	0.02	0.02	0.04	0.04	0.77	0.47	0.47	0.49
I^2^	0.55	0.57	0.56	0.54	0.55	0	0	0	0
Egger's p	0.31	0.39	0.99	0.97	0.65	0.25	0.43	0.51	0.52

Of the nine studies that explored C282Y mutation, seven studies used healthy controls, while five studies used chronic liver disease patients as controls. To clarify whether or not C282Y increased HCC in subgroups, we performed subgroup analyses between the comparison of (1) HCC and healthy controls of seven studies, (2) HCC and alcoholic LC patients of four studies, (3) HCC and viral LC patients of four studies.

**Figure 2:**
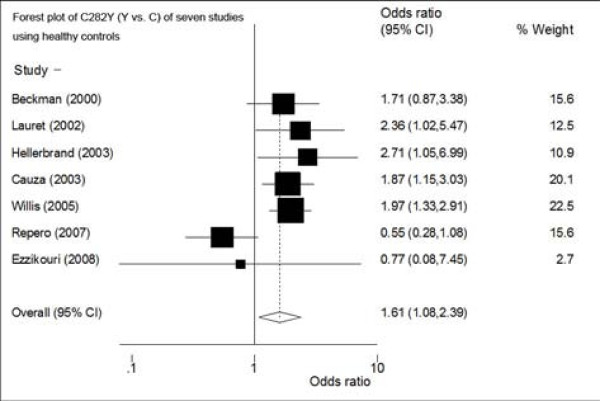
**Forest plot of the RE ORs and 95% CIs of the studies on the association between HCC and the HFE C282Y mutation (Y vs. C) of seven studies (using healthy controls)**.

**Figure 3:**
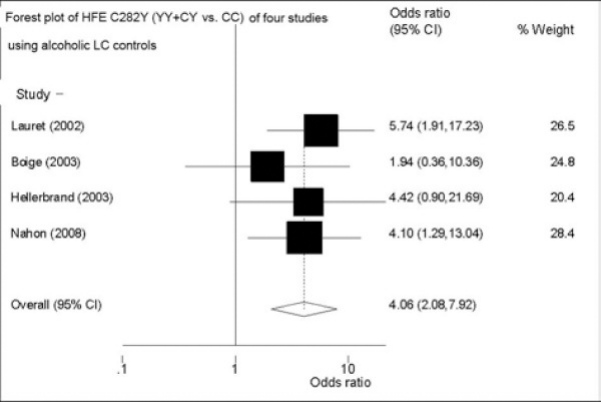
**Forest plot of the FE ORs and 95% CIs of the studies on the association between HCC and the HFE C282Y mutation (YY+CY Vs. CC) of four studies (using alcoholic LC controls)**.

**Figure 4:**
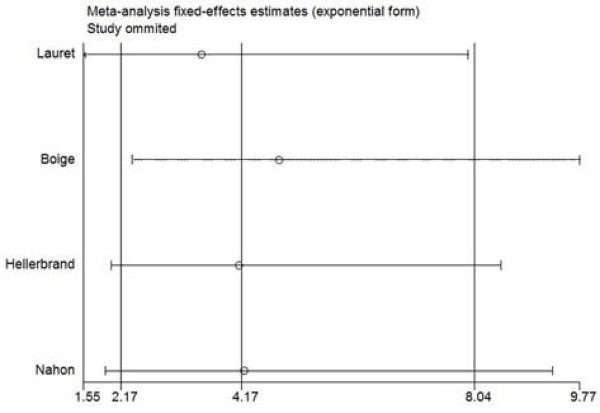
**Sensitivity analysis of the association of C282Y (YY+CY vs. CC) and HCC among alcoholic LC patients of four studies, in which the meta-analysis estimates were computed omitting one study at a time**. The results indicated the association was robust.

(1) When comparing C282Y polymorphisms between HCC cases and healthy controls of seven studies, allele contrast (Y vs. C) showed association with HCC: RE OR reached 1.61 (95%CI: 1.08-2.39) (figure 2) (Table 2). There was heterogeneity among studies (p for heterogeneity = 0.04, I^2 ^= 0.55). Sensitivity analysis showed that the result was also not robust (figure not shown). There was no small-study bias among the studies (Egger's p = 0.65).

(2) Four studies used alcoholic LC patients as controls. Four studies included 224 HCC patients with alcoholic LC and 380 alcoholic LC patients without HCC. Meta-analysis provided more distinct association of C282Y polymorphism with HCC among alcoholic LC patients. FE OR reached 4.06 (95%CI: 2.08-7.92, p for heterogeneity = 0.77, I^2 ^= 0) in the dominant model (Figure 3), and 3.41(95%CI: 1.81-6.41, p for heterogeneity = 0.47, I^2 ^= 0) as allele Y compared with allele C, respectively (Table 2). Sensitivity analyses of two models both gave robust results. Figure 4 showed the sensitivity analysis of the dominant model. There was no small-study bias (Egger's p: 0.25-0.43).

(3) Meta-analysis of four studies that used viral LC patients as controls (including 160 case and 203 controls) showed both dominant model and allele contrast had a non-significantly decreased risk of HCC (FE OR = 0.70, 95%CI: 0.32-1.50 and FE OR = 0.71, 95%CI: 0.34-1.50, respectively). There was no small-study bias among studies (Egger's p = 0.51 and 0.52, respectively) and no heterogeneity among studies (I^2 ^= 0) (figure not shown).

#### H63D

Eight studies (included 958 cases and 2258 controls) provided H63D genotype data. Variant D allele frequency was 16.81% (322/1916) in cases and 14.32% (657/4516) in controls, respectively.

Overall, this meta-analysis did not show H63D polymorphisms had influence on HCC occurrence. FE OR was 1.19 (95%CI: 0.90-1.58, p for heterogeneity = 0.01, I^2 ^= 0.60) and1.08 (95%CI: 0.83-1.39, p for heterogeneity = 0.01, I^2 ^= 0.61) in the dominant model and allele contrast model, respectively (figure not shown). There was no small-study bias among studies (Egger's p = 0.62 and 0.34, respectively). We also performed subgroup meta-analysis according to the characteristics of controls (healthy controls and chronic liver diseases controls), but all genetic models did not show evidence of associations with HCC (detailed data not shown).

The statistic power is an important issue on gene-disease association study. As for the association between C282Y polymorphism with HCC among alcoholic LC patients, we used the allele contrast (Y vs. C) data to calculate the power. According to the parameters (frequency of the mutation allele Y in the controls was 0.022, case number was 224 and control number was 380, pooled OR was 3.41, α = 0.05), PS software gave a power of 0.82, which was satisfactory. However, power of the association study on HCC and viral LC patients (160 cases and 203 controls, frequency of variant allele Y = 0.05, pooled OR = 0.71, α = 0.05) was very low (0.09).

By using the results of the meta-analysis (ORs and 95%CIs) and the knowledge of the epidemiological data of HCC (prior probability) in different populations, we derived FPRP to assess the reliability of the association. OR of allele contrast (Y vs. C) equaled 3.41 (95%CI: 1.81-6.41) in the subgroup analysis of four studies using alcoholic LC controls. If the prior probability of developing HCC in alcoholic LC patients is assigned at 0.01, then FPRP was 0.03 (<0.20).

Given that mutation allele Y of C282Y is a risk factor of HCC, we further calculated PAR and its' 95%CI in all populations and in alcoholic LC patients. According to the formula from Bruzzi, PAR of allele Y is 2.48% (95%CI: 1.30%-3.65%) and 5.12% (2.57%-7.67%) in all populations and in alcoholic LC patients, respectively.

## Discussion

HH is a common genetic disease in European populations that causes an inappropriately high absorption of iron, leading to the progressive accumulation of iron in the liver. The two missense mutations C282Y of the HFE gene explain most of the cases of HH, a condition characterized by hepatic iron overload. Liver iron accumulation leads to reactive oxygen species formation in the liver, thus causing oxidative stress. It has been shown that the wild-type HFE protein forms a stable complex with the transferrin (TF) receptor (TFR), thereby reducing its affinity for TF [32], whereas the HFE 282Tyr mutation almost completely prevents the formation of a complex between the mutant HFE protein and the TFR, allowing a high-affinity TF binding to the TFR. This binding results in an increased cellular uptake of iron. A second missense mutation in the HFE gene, H63D, is found in about 4% of patients with HH, but its role in iron overload is still debated [6].

It has been reported that HCC occurred more in HH patients than in normal populations in some cohort studies [4,33,34]. However, there are also opposite reports that HH had low penetrance and did not increase the risk of HCC [20,35,36].

From the late 1990s, many researchers have explored the relationship between these two mutations and HCC susceptibility by using case-control or cohort studies [7-9,11-19,30]. In 2007, Christina Ellervik and her colleagues [37] performed a meta-analysis to examine associations between C282Y and H63D mutations with HCC. The meta-analysis included nine studies and reported that C282Y homozygotes YY versus CC obtained an odds ratio of 11 to HCC occurrence. However, the sample sizes of many studies included in that meta-analysis were too small, leading to low statistical power. From then on, several articles about HFE mutations and HCC have been published. We selected nine eligible studies including 1102 cases and 3766 controls to conduct an updated meta-analysis.

Because HH is more frequent in northern European populations, the studies on HFE gene mutations and HCC are mainly come from European ethnicities. In this meta-analysis, eight studies were come from Europe and one from Africa. So, the analysis results may be mainly applicable to European populations and it warrants to be studied in other ethnicities. In this meta-analysis, the frequency of C282Y YY homozygotes was 0.42% (16/3766), and the frequency of CY heterozygotes was 9.32% (351/3766) in all control subjects. The genotype distribution was consistent with the dbSNP data. H63D genotype distribution was 2.66% (60/2258) and 23.78% (537/2258) for DD homozygotes and HD heterozygotes in controls, respectively.

As to C282Y, the ORs of allele contrast (Y vs. C) in the six studies [8,10-12,15,31] were larger than 1.0. Among the six studies, four studies [8,10-12] reported a significant association between HCC and the C282Y polymorphism (ORs > 1.0, 95%CIs did not include 1.0). Because the frequency of the homozygous mutation of C282Y is very low, and a large proportion of C282Y homozygotes had been diagnosed with HH and received treatment, such as venesection before developing LC or HCC, the conclusion that YY homozygotes increased HCC risk may have little clinical value. Thus, we only explored the dominant model and allele contrast in this meta-analysis. This meta-analysis proved that C282Y mutation was associated with HCC in European populations, especially in alcoholic LC patients but not in viral LC patients. This result is consistent with the results of three previous studies [8,15,38], and it may implicate that the hepatocarcinogenesis of alcoholic LC and viral LC is different and warrants further study. Some studies explored the role of gender in the influence of the relationship between HFE gene and HCC [10,14,34] and found that C282Y homozygotes YY mutation increased the risk of HCC in male patients. One English study [10] reported that male C282Y homozygotes were more likely to be diagnosed with HCC (OR = 14, 95%CI: 5-37), and the penetrance of the C282Y homozygous genotype, with respect to HCC, was between 1.31% and 2.1% for males and zero for females. Another study [36] reported that C282Y homozygote males had a relative risk (RR) of about 23 for HCC occurrence, and the penetrance, with respect to HCC, was 5.56%. As there were few studies that provided concrete gender subgroup genotype values, we could not make a pooled analysis.

From the pooled genotype data, we could assess the statistical power under various subgroup analyses using PS software [27]. We noted that the power was satisfactory except when comparisons were performed between HCC cases and viral LC patients (power = 0.09). This indicated that the null association of C282Y and HCC when compared in HCC cases and viral LC cases should be taken with caution and that it warranted further study in a larger scale. FPRP is a valuable criterion to assess whether or not a positive discovery came about by chance. We used FPRP to assess the positive association attained by this meta-analysis. The association between C282Y (Y vs. C) and HCC attained by subgroup analysis of four studies using alcoholic LC patients as controls was proved to be reliable (FPRP = 0.03).

Population-attributable risk (PAR) is a valuable parameter to assess the influence of risk factors on disease occurrence. The PAR of the variant allele Y of C282Y among alcoholic LC patients was 5.12% (95%CI: 2.57%-7.67%). This result suggested that the role of C282Y polymorphism on HCC occurrence was modest.

## Conclusions

This meta-analysis proved that C282Y mutation was associated with HCC in European alcoholic LC patients. The role of C282Y polymorphism on HCC occurrence was modest. The association of this polymorphism and HCC is warranted further studies in large scale including diverse ethnicities. The molecular mechanism of the different effect of C282Y on alcoholic LC and viral LC, with respect to HCC occurrence, also merits further studies. This meta-analysis did not find association of H63D mutation with HCC.

## Competing interests

The authors declare that they have no competing interests.

## Authors' contributions

FJ participated in the design of the study and performed the statistical analysis. XZS conceived the study, participated in its design and coordination work, and helped draft the manuscript. LSQ helped search articles and revised the draft. All authors read and approved the final manuscript.
